# COVID-19: a unique learning opportunity if the well-being of learners and frontline workers is adequately supported

**DOI:** 10.1007/s40037-020-00596-y

**Published:** 2020-05-19

**Authors:** Paul L. P. Brand

**Affiliations:** 1 Isala Academy, Department of Medical Education and Faculty Development, Isala Hospital, Zwolle, The Netherlands; 2 Lifelong Learning Education and Assessment Research Network (LEARN), University of Groningen and University Medical Centre, Groningen, The Netherlands

In the past few months, the COVID-19 pandemic has had an unprecedented impact on the affected societies. Governments worldwide have implemented various ‘lockdown’ approaches to ‘flatten the curve’ and avoid overburdening the healthcare system, particularly intensive care units. This affects the entire world population, either through direct health effects or by the ensuing global economic crisis which is now unfolding. Undoubtedly, *Perspectives on Medical Education *readers have witnessed the effects of the COVID-19 pandemic on the medical education community. Most medical faculties and hospitals responded to the impending or emerging healthcare crisis by upscaling the COVID-19 care. Triage tents and separation of ‘dirty’ and ‘clean’ care were instituted at emergency rooms, ICUs expanded into operating theatres and recovery rooms. All ‘non-essential’ care was downscaled: operations postponed, follow-up visits cancelled or rescheduled into video consultations, hospital staff urged to work from home as much as possible. A major operation carried out under enormous time pressure in most hospitals—everyone wanted to avoid the healthcare system overflow that Wuhan in China and Lombardy in northern Italy experienced. In this pressure cooker, most hospitals and medical faculties did not consider providing medical education to students, clinical clerks, and residents a priority. Residents from non-COVID departments were mobilised and put to work at COVID-19-cohort wards or ICUs, after a crash course in ventilatory support and diagnosing and managing severe pneumonia; medical students and clinical clerks were sent home, waiting for the storm to pass.

There are exceptions to this rule. At Aalborg University in Denmark, for example, final year medical students were trained and put to work as temporary residents, ventilatory therapy assistants or nursing assistants, while teaching to other students was rapidly transferred to a digital platform [1]. This approach does justice to two important considerations: that this pandemic constitutes a unique learning opportunity for medical students, residents and licensed healthcare workers, and that medical students want to help, they want to *do* something, to contribute meaningfully to the care of these patients or to support the clinicians in the frontline [2].

What wisdom should prevail here? Should clinical teaching units send clinical students and clerks home to allow them to focus on dealing with the COVID-19 patient surge, to save precious protection equipment, to reduce the risk of virus transmission by and to students? Or should medical students be welcomed and encouraged to contribute, because they are available, willing, and eager to help and learn? In this issue of *Perspectives*, a medical student, two clinical teachers and a medical education scientist from Switzerland, Austria and Canada take a nuanced look at this issue [3]. After a balanced discussion of the arguments for both positions, they propose that medical students should be welcomed to contribute to the healthcare needed for this crisis, and learn from it. They stress that supervision, feedback and evaluation of these students should not be abandoned even when faculty is extremely busy and tired. They eloquently make the point that providing medical education during this crisis is just as vital as providing the care itself—the word ‘doctor’ is, after all, derived from the Latin verb ‘docere’, teaching.

In addition to Klasen et al.’s plea to continue to provide optimal, ethical and timely medical education to students and residents during this crisis, I would like to highlight the importance of supporting the emotional and psychological well-being of these learners and their supervisors in these trying times. There is ample evidence from previous epidemics that healthcare crises can be traumatic experiences with a major impact on healthcare workers’ emotions, stress levels, and mental health [4–8]. One to two years after the 2003 SARS epidemic, for example, burnout rates were considerably higher in healthcare workers from hospitals with large numbers of SARS patients than in hospitals with no SARS cases [9]. Reports on the psychological impact of the COVID-19 pandemic on healthcare workers are now appearing from China, with stress-related symptoms such as depression, anxiety and insomnia in >30% [10], and moderate-severe mental health problems in 28% of healthcare workers in the Wuhan area where the COVID-19 epidemic struck most severely [11]. In my own hospital, which went from 2 to 80 test-proven COVID-19 patients within one week, I heard some impressive experiences from the residents in the COVID cohort and ICU wards. They struggled with the sheer volume of the patient load, with the new and unknown disease with its unpredictable and capricious course, with the lack of effective therapeutic options, and with the high mortality rate. They struggled even more with the limitations imposed by their protective equipment, which hindered them from providing empathic care, comfort and emotional support to these very sick, scared and lonely patients. They had to learn how to break bad news to patients’ families over the phone. This was something they had never done before, they learned each and every day, but it was physically and emotionally draining. They also highlighted, however, the incredible team spirit of everyone involved—junior doctors, senior supervisors, nurses, allied health professionals, cleaners, hospital management. They felt proud to be on a team so dedicated to provide the care, make it happen, just do it. Based on our existing peer support system and the experience from other Dutch hospitals which handled the first surge of patients, we instituted daily debriefing sessions and peer support to the doctors involved in COVID care. This was well attended and highly appreciated: it helped residents to realise that their emotions were normal and shared by others, including their supervisors.

Peer support has been shown to reduce the risk of long-term stress-related symptoms after a health crisis like the SARS epidemic [4], or after patient safety incidents or malpractice suits [12]. Klasen and coworkers’ call for careful support for medical students in the COVID-19 crisis is therefore timely and justified. It is likely that healthcare workers in a pandemic crisis can be supported by meeting their basic daily needs (e.g., food and drinks delivered on the ward, protecting break time), ensuring high-quality communication with reliable messages, and robust psychosocial support promoting resilience and self-care, individual and group debriefing, and further psychological support on an as-needed basis [13]. Providing such support to medical students and residents is not a luxury—it is a requirement to help them to remain fit for practice, and to allow them to benefit most from this unique learning opportunity.
